# Molecular recognition of RhlB and RNase D in the *Caulobacter crescentus* RNA degradosome

**DOI:** 10.1093/nar/gku1134

**Published:** 2014-11-11

**Authors:** Jarrod E. Voss, Ben F. Luisi, Steven W. Hardwick

**Affiliations:** Department of Biochemistry, University of Cambridge, Tennis Court Road, Cambridge CB2 1GA, UK

## Abstract

The endoribonuclease RNase E is a key enzyme in RNA metabolism for many bacterial species. In *Escherichia coli*, RNase E contributes to the majority of RNA turnover and processing events, and the enzyme has been extensively characterized as the central component of the RNA degradosome assembly. A similar RNA degradosome assembly has been described in the α-proteobacterium *Caulobacter crescentus*, with the interacting partners of RNase E identified as the Kreb's cycle enzyme aconitase, a DEAD-box RNA helicase RhlB and the exoribonuclease polynucleotide phosphorylase. Here we report that an additional degradosome component is the essential exoribonuclease RNase D, and its recognition site within RNase E is identified. We show that, unlike its *E. coli* counterpart, *C. crescentus* RhlB interacts directly with a segment of the N-terminal catalytic domain of RNase E. The crystal structure of a portion of *C. crescentus* RNase E encompassing the helicase-binding region is reported. This structure reveals that an inserted segment in the S1 domain adopts an α-helical conformation, despite being predicted to be natively unstructured. We discuss the implications of these findings for the organization and mechanisms of the RNA degradosome.

## INTRODUCTION

In all life examined thus far, RNA molecules play multifaceted roles in the controlled expression of genetic information, and their actions contribute to organism fitness under myriad and often rapidly varying environmental conditions. In many bacterial species these roles of RNA are mediated by machinery of RNA processing and decay, whose components are functionally, and often physically, linked (1). One of the key enzymes of RNA processing and decay in numerous bacterial species is the endoribonuclease RNase E. In addition to its well characterized hydrolytic nuclease activity, RNase E also acts as a molecular hub that co-ordinates the formation of a multi-enzyme assembly termed the RNA degradosome. The paradigm *Escherichia coli* RNA degradosome has been the focus of several studies, and has been shown to be composed of RNase E, the DEAD-box RNA helicase RhlB, the glycolytic enzyme enolase, and the phosphorolytic exoribonuclease polynucleotide phosphorylase (PNPase). Several other proteins have been shown to associate dynamically or at sub-stoichiometric levels (2).RNase E of *E. coli* can be divided into two functionally distinct halves. The globular N-terminal domain (*Ec*NTD; residues 1–529) contains the catalytic site and is highly conserved in the extensive family that includes RNase G paralogues (3). The crystal structure of the RNase E catalytic domain from *E. coli* reveals a homo-tetrameric organization, formed by a dimer-of-dimers with scissors-like internal quaternary structure (4). The protomer of *Ec*NTD can be divided into large and small domains, which are connected by a pair of conserved CPxCxGxG motifs that co-ordinate a zinc ion (5). Within the large domain, four sub-domains can be further assigned based on function and similarity to homologous structural folds, namely RNase H, DNase I, S1 and 5′-sensor. The catalytic domain of RNase E is well conserved at the amino acid sequence level throughout bacteria and also in homologues found in the chloroplasts of some plants. However, the only significant deviation from conservation of this domain is a highly diverse insertion within the S1 domain in members of the α-proteobacteria and plants. The S1 domain insertions vary in sequence and length, from ∼50 to 150 amino acids in differing organisms. The only seemingly consistent properties of the insertion are the location within the S1 domain, a high proportion of charged amino acids (Supplementary Figure S1A) and predicted propensity for structural disorder (Supplementary Figure S1B). Although the role of this insertion is not understood, its functional importance is suggested by the finding that deletion of the insertion from RNase E of *Arabidopsis thaliana* greatly reduced the enzyme's ribonuclease activity (6).

In *E. coli*, the C-terminal domain of RNase E (*Ec*CTD; residues 530–1061) is predicted to be natively unstructured but is punctuated by short regions with structural propensity, which are the sites for interaction with the other components of the RNA degradosome (1,2). Unlike the highly conserved NTD, the CTD of RNase E is highly divergent even amongst species that are closely related, and this may reflect the diversity of proteins able to interact with RNase E in different organisms. We have recently characterized the RNA degradosome in the α-proteobacterium *Caulobacter crescentus*, and have shown that the canonical components of this assembly are RNase E, RhlB, aconitase and PNPase. Additionally, we were able to identify segments of RNase E corresponding to recognition sites for aconitase and PNPase (7). Subsequently, we solved the x-ray crystal structure of the PNPase component of this assembly, and revealed how RNase E interacts with PNPase in *C. crescentus* (8).

In this study, we expand on our previous characterization of the *C. crescentus* RNA degradosome, and show that the essential exoribonuclease RNase D also forms part of this assembly. We demonstrate that the helicase component of the degradosome, RhlB, interacts directly with a portion of the catalytic domain of RNase E and the contact site is mapped to the C-terminal extension (CTE) of the helicase. The proximity of the helicase to the RNA binding site of RNase E is likely to be of mechanistic importance for RNA degradation and processing. Furthermore, we have determined the crystal structure of a fragment of the *C. crescentus* RNase E catalytic domain encompassing the helicase-binding region, and show that a significant portion of the S1 insertion adopts an α-helical structure despite being predicted to be natively unstructured. The structure of the inserted region in the S1 domain of *C. crescentus* RNase E is not compatible with the mechanism of RNA binding seen in the crystal structure of the *E. coli* enzyme, and we suggest that a conformational transition is required in the putative RNA binding cleft of the enzyme to accommodate substrates. These findings illustrate how the RNA degradosome has undergone convergent evolution to recruit a helicase while undergoing divergent evolution in recruiting species-specific components and the binding of RNA substrates at the active site.

## MATERIALS AND METHODS

### Cloning, expression and purification of ANCHOR predicted recognition domains and use in GST affinity pull downs

Protein recognition segments in RNase E predicted by the program ANCHOR (residues 760–803 and 820–876; see ‘Results’ section) were amplified from genomic DNA using the primers RNE760–803.F (5′-CGGGATCCGCGCCGGTCGCCGAGATGACCTCG-3′), RNE760–803R (5′-CGCTCGAGTTAGACTTCCCGCAGTTCGACCCAAAC-3′), RNE820–876.F (5′-CGGGATCCGCGACTGAAACGTCCGTCGAAGCG-3′) and RNE820–876.R (5′-CGCTCGAGTTACGGTTGGGCCTCCTCGACGGCTTC-3′) and ligated into the expression vector pGEX-6p1 via BamHI and XhoI restriction sites, to generate N-terminal Glutathione S-transferase (GST) tagged versions of the predicted protein recognition sites. The GST fusion proteins were expressed in the *E. coli* strain BL21(DE3), and the proteins were purified by glutathione sepharose affinity chromatography followed by size exclusion chromatography using a superdex 200 column (GE Healthcare). For pull down experiments, ∼0.5 mg of purified GST fusion protein was coupled to 100 μl of glutathione sepharose resin (GE Healthcare). The protein coupled resin was incubated at 4°C for 2 h with lysate prepared from cells pelleted from a 1 l culture of *C. crescentus* grown to mid-log phase in peptone-yeast (PYE) medium at 30°C. The glutathione sepharose was harvested, washed with pulldown (PD) buffer (20-mM Tris–HCl pH 7.5, 200-mM NaCl) and bound proteins were eluted with PD buffer supplemented with 50-mM reduced glutathione, before being visualized by sodium dodecyl sulphate-polyacrylamide gel electrophoresis (SDS-PAGE).

### Cloning, expression and purification of RNase D, and use in Nickel affinity pull down

The RNase D gene (cc1704) was amplified from *C. crescentus* (CB15) genomic DNA using the primers ccRND.F (5′-CGGAATTCGATCAAGCTGATCACCACCACCGCC-3′) and ccRND.R (CGAAGCTTTCAATCGTTCTTCGGGGGCGCGACGCACC-3′) and ligated into the expression vector pETDuet-1 via EcoRI and HindIII restriction sites to generate an N-terminal Hexa-histidine tagged protein construct. The N-terminal 6xHis-tagged protein was overexpressed in BL21(DE3) cells grown at 20°C overnight, and then purified by Ni^2+^ affinity chromatography followed by size exclusion chromatography using a superdex 200 column (GE Healthcare). For pull down experiments, ∼0.5 mg of purified RNase D was coupled to a His-select spin column (Sigma-Aldrich). Lysate from a 250 ml culture of *C. crescentus* grown to mid-log phase in PYE medium at 30°C was then passed over the spin column resin and washed with PD buffer. Bound proteins were eluted with PD buffer supplemented with 500-mM imidazole, before being visualized by SDS-PAGE.

### Cloning, expression and purification of *C. crescentus* RNase E catalytic domain and the S1/5′-sensor sub-domains

An N-terminally 6xHis tagged *C. crescentus* RNase E NTD construct (*Cc*NTD_1–575_) was generated to aid protein expression and purification. The DNA sequence encoding amino acid residues 1–575 of RNase E from *C. crescentus* was amplified by polymerase chain reaction (PCR) from pGEX-CcNTD plasmid (7) using the primers CcNTDhis.F (5′-CGCATATGTCGAAGAAGATGCTGATCG-3′) and CcNTDhis.R (5′-GCGGATCCTTATTCTTCTTCCTCGTCGTCGTA-3′). The PCR product was ligated into pET-15b using the NdeI and BamHI restriction sites. *Cc*NTD_1–575_ was overexpressed in BL21(DE3) cells grown at 37°C and then purified by Ni^2+^ affinity and heparin chromatography before dialysis into NTD buffer (20-mM Tris pH 7.5, 500-mM NaCl, 5-mM MgCl_2_, 50-mM L-Arginine, 50-mM L-Glutamic acid). The protein purity of *Cc*NTD_1–575_ was estimated by SDS-PAGE to be >98% and the specimen was free of nucleic acid, as judged by the ratio of absorbance 260/280 nm. The proteolytically liberated fragment containing both the S1 and 5′-sensor sub-domains (*Cc*NTD_1–274_) was identified by matrix-assisted laser desorption/ionization (MALDI) mass spectrometry to contain residues 1–274. This region was cloned into the first site of the pET-DUET vector (Novagen) for co-expression with RhlB in the second site using the primers CcRNE_DUET_f (5′-GCGAGCTCGATGTCGAAGAAGATGC-3′) and CcRNEfrag_DUET_r (5′-GGATCCAAGCTTAGCGTTTGACGAGGTCTTCTTCCTCG-3′). The *Cc*NTD_1–274_–RhlB complex was expressed and purified as above.

### Analytical size exclusion chromatography

Approximately 50-nmol *Cc*NTD_1–575_ (monomer) was mixed with 65-nmol RhlB in a total volume of 800 μl in NTD buffer and incubated at 4°C for 1 h. The mixture was then loaded onto a superdex 200 16/60 (GE Healthcare) gel filtration column that was pre-equilibrated in NTD buffer. In the case of the *Cc*NTD_1–274_–RhlB complex, the two partner proteins were co-expressed and purified as a complex (as described above) before size exclusion chromatography.

### Analytical ultracentrifugation

Interference-based sedimentation velocity experiments were performed in a Beckman model XL-I analytical ultracentrifuge using a 4-hole An-60 Ti rotor. Double-sector quartz cells were loaded with 380 μl of sample (*Cc*NTD_1–575_ or *Cc*NTD_1–274_–RhlB) at 2 mg/ml and 400 μl of reference (NTD buffer in the case of *Cc*NTD_1–575_ or 20-mM Tris pH 7.5, 200-mM NaCl for *Cc*NTD_1–274_–RhlB). Experiments were conducted at 20°C using a rotor speed of 40 000 rpm for *Cc*NTD_1–575_ and 50 000 rpm for *Cc*NTD_1–274_–RhlB. Solvent density, solvent viscosity and estimates of the partial specific volume of the proteins were calculated using SEDNTERP (9). Initial scans were carried out at 3000 rpm to determine optimum radial positions for the experiments. Sedimentation velocity data were fitted to a continuous molar mass [c(M)] model using the programme SEDFIT (10).

### Crystallization, x-ray diffraction data collection, processing and model building

Crystals of the proteolytic fragment of *Cc*NTD_1–575_ appeared after three days in 0.1-M Tris pH 8.5, 10% (v/v) Ethanol. For x-ray data collection, the crystals were transferred to reservoir solution containing 20% (v/v) glycerol and directly flash-frozen in liquid nitrogen. Intensity data were collected at 100 K at the Diamond Light Source I-24 beamline with x-rays at wavelength of 0.9536 Å using a PILATUS detector. Diffraction datasets were processed and scaled using iMOSFLM (11) and SCALA (12). A model was obtained by molecular replacement using PHASER (13) with the S1 and 5′-sensor domains from *E. coli* RNase E (*Ec*NTD) structure (PDB ID: 2C0B) as the search model. Structural refinement was performed using REFMAC5 (14) and iterative model building using COOT (15). The final model co-ordinates of *Cc*NTD_1–274_ have been deposited at the protein data bank, with the PDB ID: 4OXP.

## RESULTS

### Identification of RNase D as a component of the *C. crescentus* RNA degradosome

**Figure 1.**
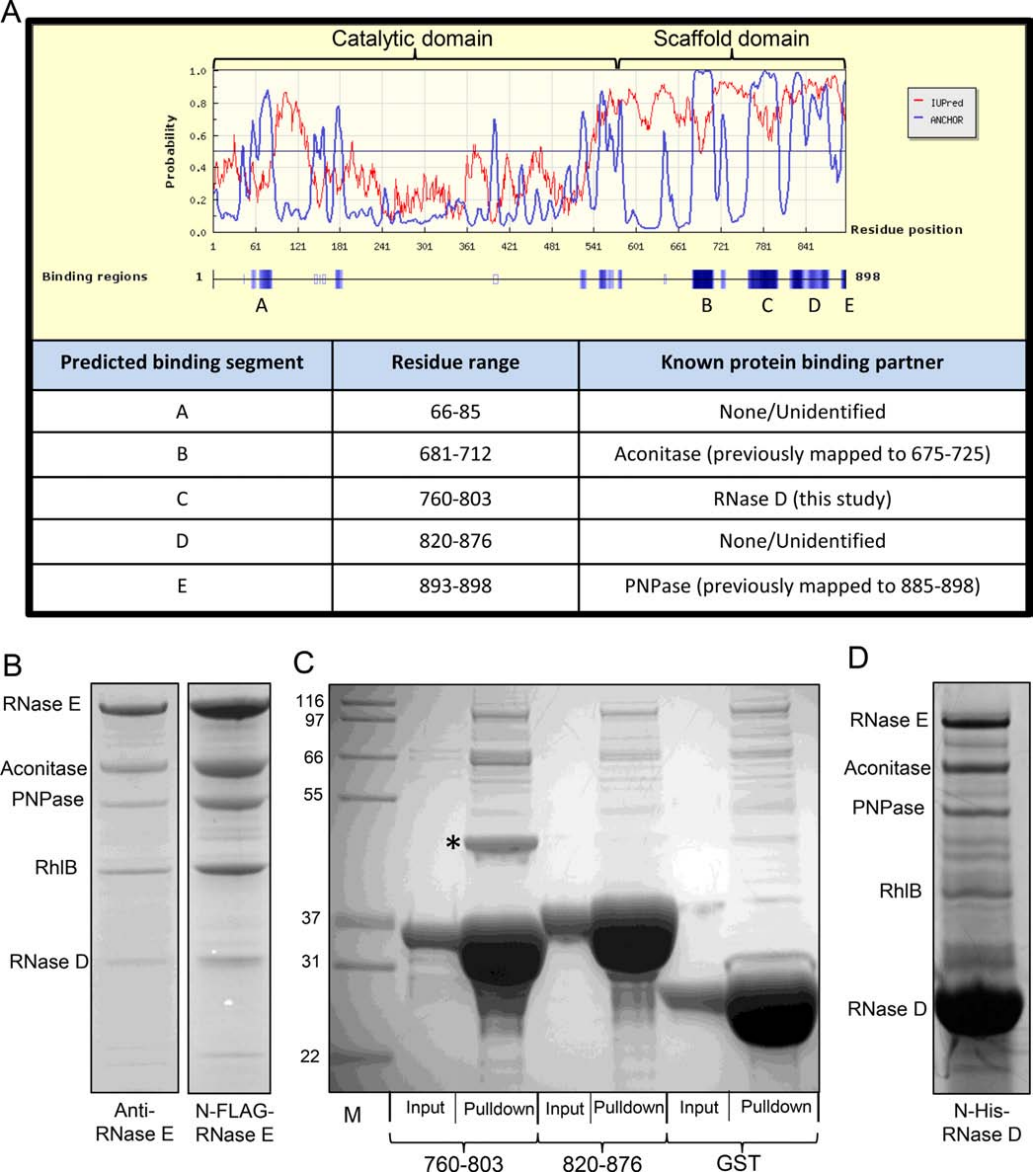
Small recognition motifs in *Caulobacter crescentus* RNase E. (**A**) ANCHOR prediction (blue line) of protein–protein interaction sites within RNase E (labelled A–E). The red line corresponds to the IUPred prediction of intrinsically unstructured regions. Bottom, a table summarizing the ANCHOR predicted protein-binding sites and known protein binding partners. The ANCHOR maxima at residue 180 and 550 correspond to regions that are not solvent exposed and are involved in intra-domain interactions respectively in the *Escherichia coli* crystal structure. (**B**) Co-immunopurification of *C. crescentus* RNA degradosome via anti-RNase E antibody, or N-terminally FLAG tagged RNase E. (**C**) Pull down experiment with GST fusions of ANCHOR predicted protein binding segments, or GST alone. RNase D is marked with an asterisk. (**D**) Reciprocal pull down experiment using 6xHis-tagged RNase D as bait. Controls are shown in Supplementary Figure S3.

We have previously identified the canonical components of the *C. crescentus* RNA degradosome by co-immunopurification, and have shown that this complex is comprised of RNase E (cc1877), the DEAD-box protein cc1847, the exoribonuclease polynucleotide phosphorylase (PNPase/cc0034) and the tricarboxylic acid cycle enzyme aconitase (cc3667) (7). The binding sites for PNPase and aconitase in RNase E were identified by sequence homology and secondary structure prediction (7,8,16), however, we were unable to find a site of interaction for RhlB. To explore further potential interaction sites, the *C. crescentus* RNase E sequence was analysed by the program ANCHOR (17) (Figure 1A). ANCHOR identifies regions of low secondary structure within a given amino acid sequence and predicts segments that have the capability to interact with a globular protein partner. The ANCHOR predictions for protein interaction sites concurred with previously characterized aconitase and PNPase binding sites (residues 681–712 and 893–898 respectively, Figure 1A). Additionally, ANCHOR identified a potential protein–protein interaction site within the catalytic domain of RNase E (residues 66–85), and two further sites within the non-catalytic CTD (residues 760–803 and residues 820–876).

To test whether either of the two predicted CTD segments were indeed protein interaction sites, N-terminal GST fusions of the individual sites (RNase E_760–803_ and RNase E_820–876_) were used as bait in pull down experiments after incubation with *C. crescentus* cell lysate (Figure 1C). The fusion construct containing RNase E_820–876_ did not significantly enrich any potential protein partners from the cell lysate compared to the negative control of GST alone. However, the construct containing RNase E_760–803_ was able to interact with one predominant protein of ∼45 kDa in the pull down. MALDI mass spectrometry analysis identified the 45-kDa band as ribonuclease D (RNase D–cc1704).

Previously, co-immunoprecipitation of RNase E under stringent conditions (7) yielded a band of 45 kDa that may correspond to RNase D, however another band of ∼45 kDa was present in the negative control experiment. As such, this band was not investigated further at the time. To address the identity of the 45 kDa band, we conducted two further co-immunopurification experiments, one with an antibody raised against the S1 insert of RNase E as described previously, and the second using an anti-FLAG monoclonal antibody for a strain with a chromosomally encoded 3xFLAG tag on the N-terminus of RNase E (8) (Figure 1B). Both purification strategies isolated the degradosome assembly, showing a similar protein pattern of the known *C. crescentus* degradosome components on SDS-PAGE gels (7). Additionally, we were able to establish by MALDI mass spectrometry that the band of ∼45 kDa present in both pull down samples, corresponded to a mixture of RNase D and elongation factor Tu (TufA–cc3199). The 45-kDa band appearing in negative control pull downs of both the anti-RNase E antibody and the anti-FLAG resin was identified to contain only EF-Tu, indicating that this protein was able to bind to the immunopurification resin non-specifically (Supplementary Figure S2). This is perhaps not surprising as during rapid growth conditions EF-Tu is one of the most abundant proteins in many bacterial cells (18).

To further characterize the interaction of RNase D with RNase E, recombinant RNase D was produced with an N-terminal hexa-histidine tag and used as bait in a pull down experiment with *C. crescentus* cell lysate. Using this procedure, the known degradosome proteins (RNase E, aconitase, PNPase and RhlB) were all identified as co-purifying protein partners of RNase D (Figure 1D and Supplementary Figure S3). In addition, the oligomeric state of the recombinant RNase D protein was assessed by analytical ultracentrifugation (AUC). Unlike the *E. coli* RNase D, which is predicted to be monomeric (19), *C. crescentus* RNase D migrated as a single species of 85.9 kDa in our AUC analysis, which is in excellent agreement with the theoretical mass of a dimer of 89.3 kDa (Supplementary Figure S4).

Taken together, these results indicate that RNase D is a partner of the RNA degradosome *in vivo* and that the principal recognition site, which is sufficient to support the binary interaction, is within RNase E residues 760 to 803.

### Interaction of RhlB with the catalytic domain of RNase E

**Figure 2.**
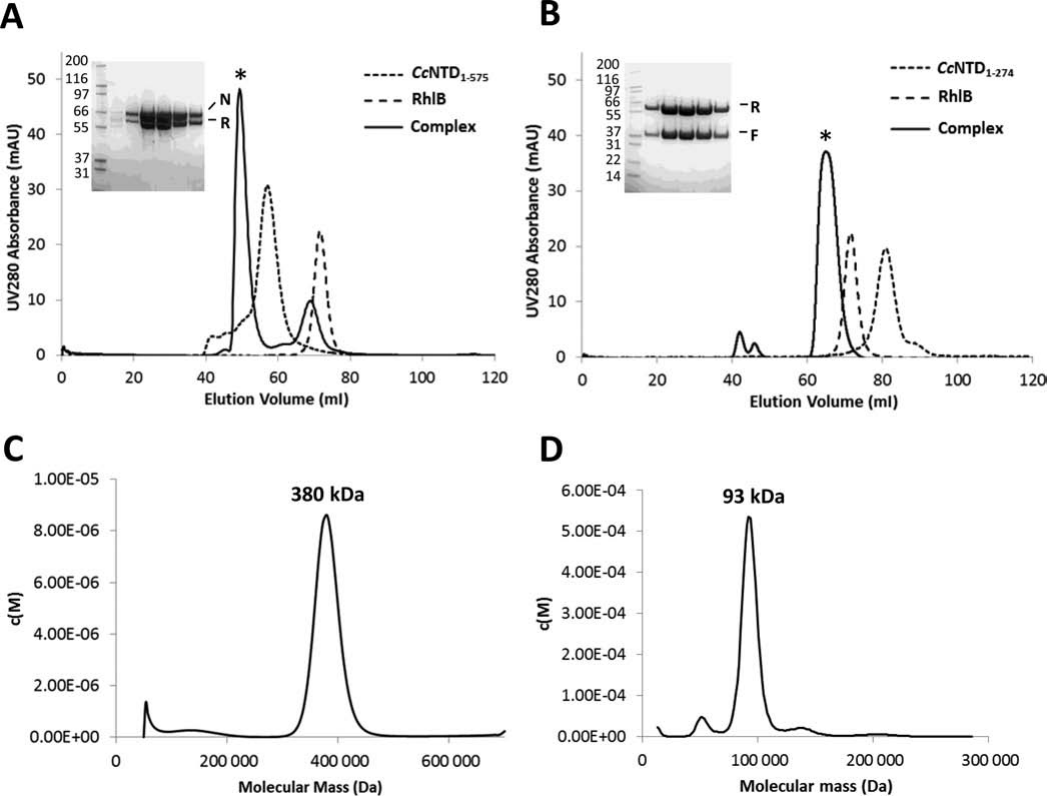
Interactions of the catalytic domain of *Caulobacter crescentus* RNase E with the DEAD-box helicase RhlB. (**A**) Size exclusion chromatography of the *Cc*NTD_1–575_–RhlB complexes. Chromatograms from three experiments (*Cc*NTD_1–575_, RhlB and a 1:1.3 molar ratio respectively) on a 16/60 S200 size exclusion column are overlaid. The SDS-PAGE gel (inset) contains fractions from the peak corresponding to the *Cc*NTD_1–575_–RhlB complex (*), with *Cc*NTD_1–575_ and RhlB labelled ‘N’ and ‘R’ respectively. The molecular weights of the standards in lane 1 are indicated (kDa). (**B**) A proteolytically liberated fragment of *Cc*NTD_1–575_ containing the S1 and 5′-sensor domains (*Cc*NTD_1–274_) forms a complex with RhlB. As for (A), chromatograms from the three experiments are overlaid. The peak corresponding to the *Cc*NTD_1–274_–RhlB complex (*) resulted from co-expressing and co-purifying the two components. The corresponding peak fractions are shown in the SDS-PAGE gel (inset), with *Cc*NTD_1–274_ and RhlB labelled ‘F’ and ‘R’ respectively. (**C**) A continuous molecular mass distribution from analytical ultra-centrifugation sedimentation velocity analysis of the *Cc*NTD_1–575_–RhlB complex in (A). The peak value corresponds to a mass of 380 kDa. (**D**) A continuous molecular mass distribution from sedimentation velocity analysis of the *Cc*NTD_1–274_–RhlB complex in (B). The peak value corresponds to mass of 93 kDa.

Having defined the RNase D binding site within RNase E, we then turned to identify the only remaining major degradosome component with an uncharacterized binding region, RhlB. We adopted a strategy of using recombinantly expressed and purified RNase E and RhlB to characterize this interaction *in vitro*. In the course of purifying *C. crescentus* RNA degradosome proteins, we identified a proteolytic fragment corresponding to a portion of the catalytic domain of RNase E that consistently co-purified with RhlB (results not shown). To examine further if the catalytic domain of RNase E (*Cc*NTD_1–575_) could interact directly with RhlB, the purified proteins were tested for interaction by size exclusion chromatography. When the two proteins were mixed at a molar ratio of 1:1.3 (*Cc*NTD_1–575_:RhlB), a species eluted from the column earlier than either of the two individual proteins alone, with an estimated molecular weight of ∼350 kDa (Figure 2A). SDS-PAGE analysis of the fractions corresponding to the earlier eluting peak revealed it to contain both *Cc*NTD_1–575_ and RhlB (Figure 2A), indicating that the two proteins interact to form a stable complex. Further analysis of these fractions by sedimentation velocity AUC gave an estimated molecular weight for the complex of ∼380 kDa (Figure 2C, Supplementary Figure S5), which is in agreement with the estimate from size exclusion chromatography. Given that *Cc*NTD_1–575_ forms a tetramer in solution of ∼260 kDa (Supplementary Figure S6), and RhlB is monomeric in solution (Supplementary Figure S7), these results suggest a ratio of two RhlB monomers to one *Cc*NTD_1–575_ tetramer, giving a theoretical molecular weight of ∼370 kDa.

### Interaction of RhlB with a fragment of CcNTD_1–575_ containing the S1 and 5′-sensor domains

During purification of *Cc*NTD_1–575_, we noticed that the protein was susceptible to proteolysis, with a seemingly stable fragment of ∼35 kDa being readily liberated, likely due to the host proteases that are released during cell lysis. The stable fragment was identified by MALDI mass spectrometry as encompassing residues 1–274, which maps to the S1 and 5′-sensor domains of RNase E (*Cc*NTD_1–274_). Curiously, RhlB co-purified with the *Cc*NTD_1–274_ fragment, indicating that the S1 and 5′-sensor domains of RNase E harbours the RhlB interaction site (Figure 2B). To further characterize this interaction, a co-expression construct of *Cc*NTD_1–274_ and RhlB was created and the complex of the two proteins was purified and analysed by sedimentation AUC. The sample showed a single predominant species with calculated molecular mass of 93 kDa (Figure 2D, Supplementary Figure S8). This mass is in agreement with the theoretical mass of 88 kDa for a 1:1 complex between *Cc*NTD_1–274_ and RhlB.

### The C-terminal extension of RhlB interacts with the catalytic domain of RNase E

**Figure 3.**
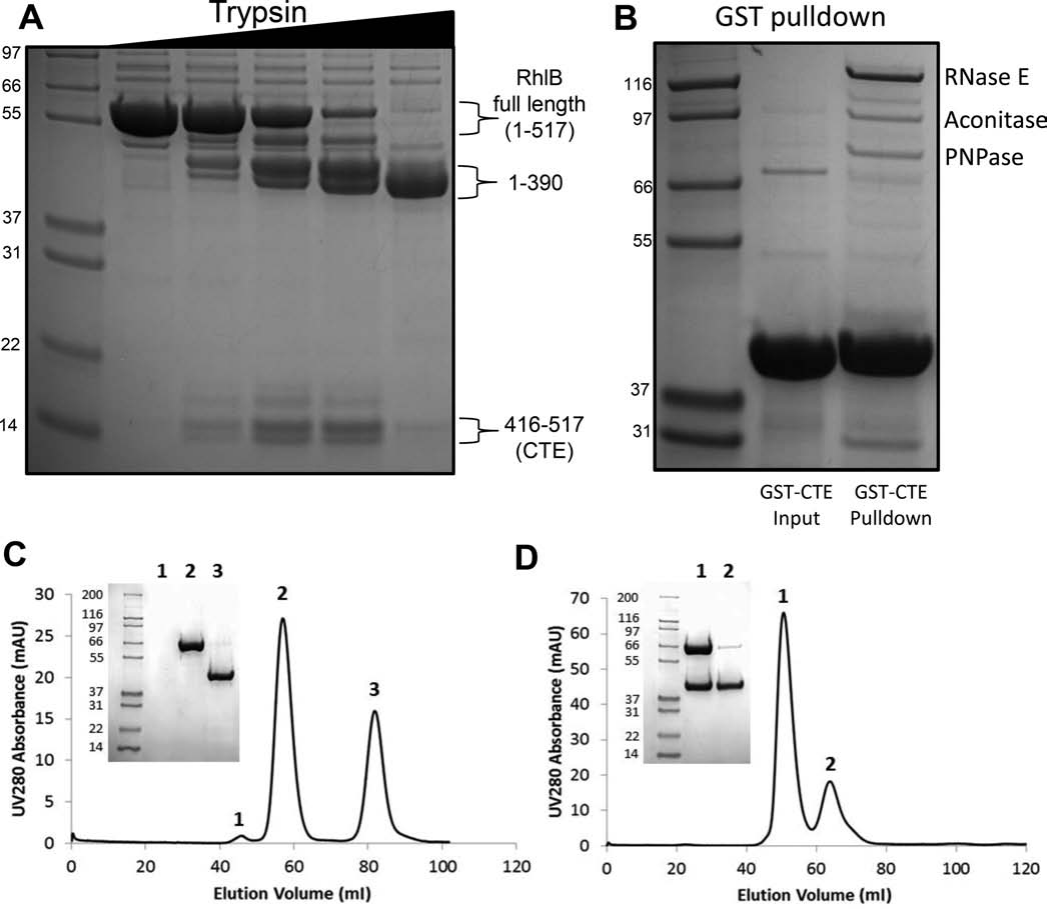
The C-terminal extension of RhlB mediates the interaction with RNase E catalytic domain. (**A**) Limited trypsin proteolysis of RhlB liberates stable fragments characterized by MALDI mass spectrometry as residues 1–390 and 416–517. (**B**) Pull down experiment with GST-RhlB_416–517_, co-purifying degradosome components are indicated. (**C**) *Cc*NTD_1–575_ was mixed with either N-RhlB (1–390) or (**D**) GST-RhlB_416–517_ with a 1:1.3 molar ratio of *Cc*NTD_1–575_ and RhlB respectively. The individual mixtures were then run on a superdex 200 16/60 gel filtration column and elution was monitored by UV absorbance at 280 nm. The numbered peaks from the elution profiles were analysed by SDS-PAGE (inset), with numbers above the gel corresponding to the peak from the profile and numbers to the left of the gel are the molecular weights of the standards (kDa).

To identify the region of RhlB required for the interaction with the catalytic domain of RNase E, stable domains of RhlB were created by limited trypsin proteolysis for subsequent use in *in vitro* binding experiments (Figure 3A). A predominant stable fragment was identified corresponding to residues 1–390. This fragment is in good agreement with the region of RhlB predicted to be structured by ANCHOR (Supplementary Figure S9) and corresponded to the conserved RecA like core of the DEAD-box enzyme (Supplementary Figures S9 and S10). An additional C-terminal fragment encompassing residues 416–517 was identified, mapping to the poorly conserved CTE of RhlB (Supplementary Figure S10). Both RhlB_1–390_ (untagged) and RhlB_416–517_ (with N-terminal GST tag) were tested for their ability to interact with *Cc*NTD_1–575_ by size exclusion chromatography. Only RhlB_416–517_ was able to form a stable interaction with *Cc*NTD_1–575_, indicating that the CTE of RhlB contains the interaction site for RNase E (Figure 3C and D). Additionally, when GST-RhlB_416–517_ was used as bait in a pull down experiment with *C. crescentus* cell lysate, the known degradosome proteins (RNase E, aconitase and PNPase) were all enriched (Figure 3B). Full length RhlB did not co-purify in this experiment, indicating that the GST-RhlB_416–517_ bait protein competes with endogenous RhlB for binding to RNase E.

### The x-ray crystal structure of the S1 and 5′-sensor domains of *C. crescentus* RNase E

**Figure 4.**
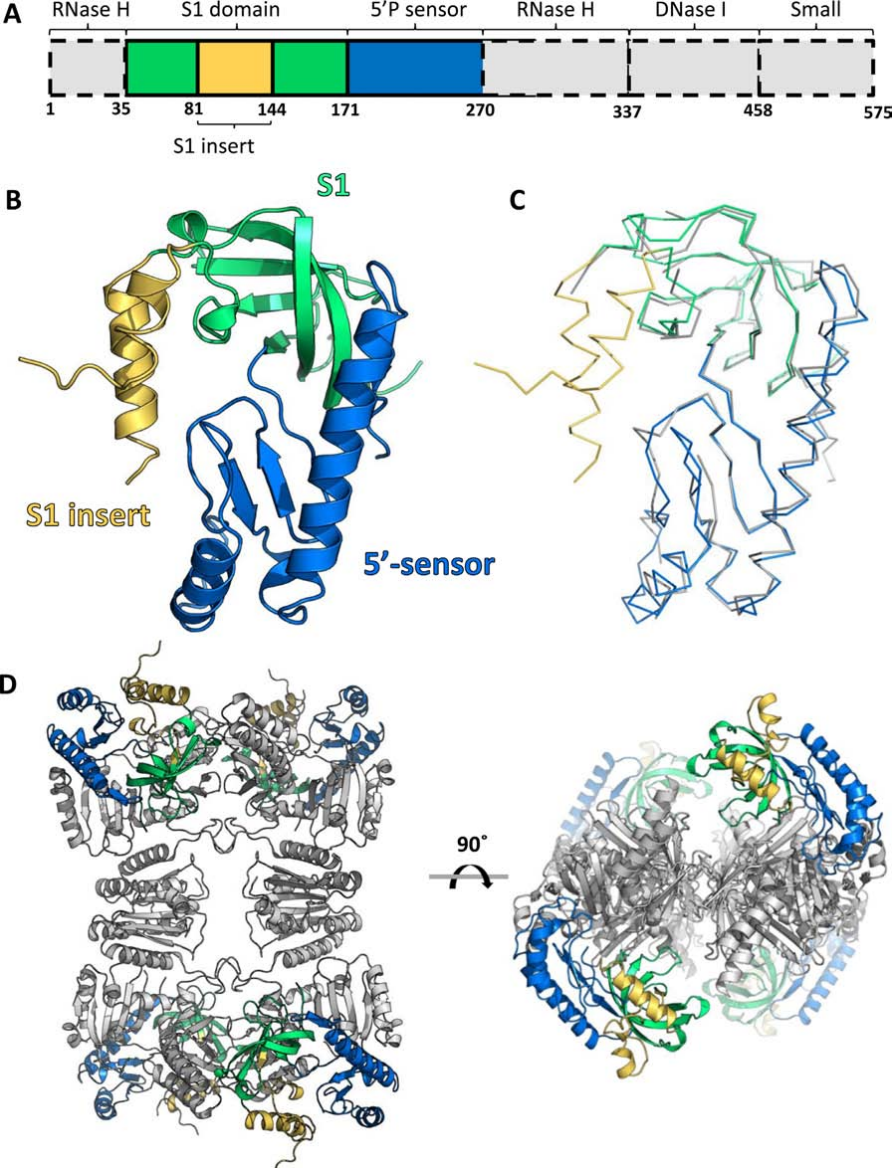
X-ray crystal structure of the *Cc*NTD_1–274_ fragment from the catalytic domain of RNase E. (**A**) A linear schematic of the *Cc*NTD_1–575_ domain architecture. RNase H, S1, 5′-sensor, DNase I and small domains are indicated along with the insertion within the S1 domain in *Caulobacter crescentus* RNase E. S1, S1 insert and 5′-sensor domains as resolved in the crystal structure of *Cc*NTD_1–274_ are coloured green, yellow and blue respectively. The regions not resolved in the crystal structure are shown in dashed boxes coloured grey. (**B**) The crystal structure of the proteolytically liberated fragment of *Cc*NTD_1–575_ (*Cc*NTD_1–274_) is shown as cartoon representation, with the S1 domain, the S1 insert and the 5′-sensor domain coloured as in (A). (**C**) The structure of *Cc*NTD_1–274_ is aligned to the equivalent portion of the crystal structure of the *Ec*NTD apo-protein (PDB ID: 2VMK) at the C-α atoms using PyMol. Both structures are shown in ribbon representation, with *Cc*NTD_1–274_ coloured as in (A), and the *Ec*NTD structure is coloured grey. The rmsd for the alignment was 0.82 Å. (**D**) *Cc*NTD_1–274_ is aligned to *Ec*NTD (PDB ID: 2C0B, rmsd at Cα = 0.82 Å) to reveal the predicted location of the former in the tetramer of *Cc*NTD_1–575_. Two views are shown after rotating 90° about the indicated axis. For *Cc*NTD_1–274_, the S1 domain, S1 insert and 5′-sensor domains are coloured as in (A). The *Ec*NTD tetramer is coloured grey.

Having established that the catalytic domain of *C. crescentus* RNase E was capable of binding directly to RhlB, we attempted to obtain structural information to rationalize the basis of this interaction. The *Cc*NTD_1–575_ construct was crystallized and x-ray diffraction data were collected to 2.1 Å resolution (Table 1). The crystal unit cell was too small to accommodate a complete *Cc*NTD_1–575_ protomer, suggesting that the protein had been proteolytically degraded prior to crystallization, leaving only a fragment of the full length *Cc*NTD_1–575_ in the crystal. Isolated sub-domains (RNase H, S1, 5′-sensor, DNase I, small domain) from the equivalent *E. coli* RNase E catalytic domain structure (*Ec*NTD, PDB ID: 2C0B) were sequentially used as search models for molecular replacement (a linear domain schematic for *Cc*NTD_1–575_ is shown in Figure 4A). Given that a stable fragment of residues 1–274 (*Cc*NTD_1–274_—see previous sections) is readily liberated during purification of *Cc*NTD_1–575_, it was unsurprising that only the adjacent S1 and 5′-sensor domains produced solutions with satisfactory scores from molecular replacement trials. A search model was prepared that combined the S1 and 5′-sensor domains from 2C0B (residues 36–214) and the solution yielded a Fo-Fc map with interpretable positive density corresponding to the *C. crescentus* S1 domain insertion segment that was absent from the *Ec*NTD search model. Several cycles of manual model building followed by refinement enabled the construction of approximately half of the S1 domain insert. Residues 95–124 in this region could not be modelled, most likely due to conformational flexibility in agreement with predictions by PONDR and ANCHOR (16,17). The final model encompasses residues 35–270 of *Cc*NTD, which corresponds to the proteolytic fragment capable of interacting with RhlB that we had observed in the previous sections (*Cc*NTD_1–274_).

**Table 1.** Crystallographic data for the fragment of the catalytic domain from *C. crescentus* RNase E (PDB ID: 4OXP)

Diffraction statistics		Refinement statistics	
Space group	P4_3_2_1_2	Rfactor	0.21
Cell dimensions		Rfree	0.25
a = b, c (Å)	62.53, 155.55	Number of reflections used	8114
Resolution (Å)	2.10 (2.21–2.10)	Total number of atoms	1524
Rmerge	0.078	Rmsd (bonds, Å)	0.019
I/σI	12.8 (2.8)	Rmsd (angles, degrees)	2.31
Completeness (%)	97.0 (98.2)		
Redundancy	5.2 (5.3)		
Number of unique reflections	18 069 (2628)		
Wilson B factor (Å^2^)	66.7		

Numbers in parentheses correspond to reflections in the high resolution bin.

**Figure 5.**
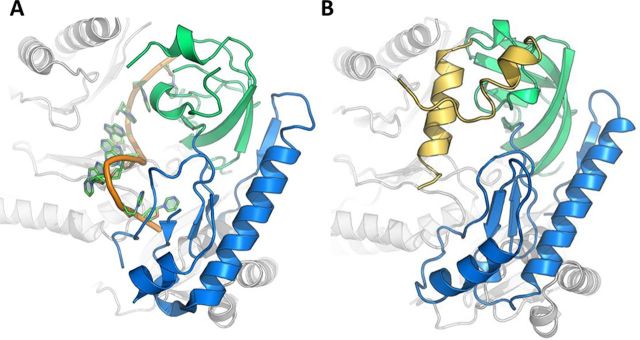
Comparison of the *Cc*NTD_1–274_ and the RNA-bound *Ec*NTD crystal structures. (**A**) Close up view of the RNA binding site in the *Ec*NTD crystal structure (PDB ID: 2C0B). The RNA backbone is coloured in orange, the S1 domain (36–118) in green and the 5′-sensor domain (119–214) in blue. (**B**) The equivalent view of the *Cc*NTD_1–274_ crystal structure aligned onto *Ec*NTD, showing the S1 insert helices in yellow (residues 97–145) protruding into the putative RNA binding site. All colouring is consistent with previous figures.

The *Cc*NTD_1–274_ structure is very similar to the equivalent portion of the *Ec*NTD structure (PDB ID: 2C0B, rmsd = 0.82 Å based on Cα alignment, Figure 4B and C), which is not surprising given the high sequence identity between the two molecules (Supplementary Figure S11). To give an indication of the probable position of *Cc*NTD_1–274_ within the tetrameric *Cc*NTD_1–575_, four copies of the *Cc*NTD_1–274_ structure are overlaid on the *Ec*NTD tetramer (Figure 4D). The portions of the S1 insert that could be modelled are on the surface of the tetramer and are solvent exposed. Although we were able to confirm that the *Cc*NTD_1–575_ construct forms a tetramer by AUC (Supplementary Figure S6), it is possible that the detailed structure may differ to that of the equivalent *Ec*NTD tetramer depicted in Figure 4D. Nonetheless, it is noteworthy that a helix from the S1 insert protrudes directly into the predicted RNA binding site of tetrameric *Cc*NTD (Figure 5). The close proximity of the S1 insert to the predicted RNA binding site would affect the recognition or binding of its targets, with possible regulatory consequences.

## DISCUSSION

Following on from our previous work that identified the major components of the *C. crescentus* RNA degradosome as RNase E, aconitase, PNPase and the DEAD-box helicase RhlB (7), we now show that the exoribonuclease RNase D is also a component of this assembly. The identification of RNase D as part of the *C. crescentus* RNA degradosome expands the functional repertoire of this complex. RNase D is classified as a member of the DEDD family of exoribonucleases, along with RNase T, oligoribonuclease and is part of a larger superfamily that includes the proofreading domains of many DNA polymerases and DNA exonucleases (20). *E. coli* RNase D plays a role in 3′ processing of numerous stable RNA species, including tRNA, 5S RNA and several other small structured RNAs (21–25). The role of RNase D in the *C. crescentus* RNA degradosome is still to be elucidated, but it is interesting to note that the enzyme has a homologue in eukaryotes, Rrp6, which is a component of the cytoplasmic exosome complex and aids in processing and degradation of RNA substrates (26). In another intriguing parallel, the core of the exosome is evolutionarily related to the degradosome PNPase. While Rrp6 interacts with the PNPase-like core of the exosome, in *C. crescentus* it appears to interact principally with RNase E. Given the role of RNase D in processing tRNAs in *E. coli* and the parallels with the exosome in eukaryotes, it seems possible that RNase D in the *C. crescentus* degradosome targets small structured RNAs and may be involved in surveillance and quality control mechanisms.

The *C. crescentus* genome contains two genes annotated as RNase D, namely, the cc1704 gene encoding the 43 kDa protein described in this study and gene cc3603, encoding a putative 23-kDa RNase D protein (Supplementary Figure S12), both of which are essential (27). It is not uncommon for genomes to encode more than one close homolog of RNase D, many of which are shortened at the C-terminus, as is the case for cc3603 (20). We did not observe any protein corresponding to the shorter RNase D in our pull down experiments, suggesting that the interaction site for the longer RNase D might lie in its CTD. The interaction site for RNase D in *C. crescentus* RNase E is not conserved in *E. coli* RNase E. This may explain why RNase D is not a component of the *E. coli* degradosome.

The interaction between RhlB and RNase E in *E. coli* has been the subject of several previous studies, and the recognition site for that helicase has been mapped to a short segment of the non-catalytic CTD of RNase E (residues 719–731) (28). However, a similar segment could not be identified in *C. crescentus* RNase E by protein sequence alignment. Instead, we show that the site of interaction is within the S1 and 5′-sensor domains of RNase E. A potential binding site within this region of RNase E is predicted by ANCHOR (residues 66–85, segment A in Figure 1A) but we have found that this segment is not sufficient for isolating RNA degradosome proteins from cell lysate.

Our size exclusion chromatography and AUC analyses indicate that in isolation *Cc*NTD_1–274_ is able to bind to RhlB at a 1:1 molar ratio. However, in the context of the tetrameric *Cc*NTD_1–575_, it appears that the stoichiometry of this complex is altered to a 2:1 ratio. Given that the tetrameric form of RNase E is expected to be the predominant form of the enzyme in the cell, we would expect the 2:1 ratio of this complex to also be found *in vivo*. Ribosome profiling data also indicate that the number of molecules per cell, based on translation levels, of RNase E and RhlB in *C. crescentus* are 3728 and 2060 respectively (29), consistent with a 2:1 complex of RNase E:RhlB. It is not clear why only two RhlB monomers can be engaged on one RNase E tetramer when there are four possible interaction sites, but perhaps the binding of the RhlB to the NTD occludes the symmetrical sites through steric hindrance. The 2:1 stoichiometry of the *Cc*NTD:RhlB complex implies that the assembly will have intrinsic asymmetry, and it is possible that this results in an inequivalence of the ribonuclease catalytic sites.

The interaction of RhlB and RNase E in the *C. crescentus* degradosome provides another example of the physical link between ATP-dependent RNA helicases/remodelling enzymes and ribonucleases. The close cooperation between RNA helicases and RNA degrading enzymes is not uncommon in evolution, with examples including the eukaryotic exosome assembly, and the non-sense mediated decay pathway (30). Given that RhlB interacts with the S1 and 5′-sensor domains of *Cc*NTD, the helicase and the active site of RNase E will be in close proximity within the degradosome assembly. Such a close interaction between the RNase E catalytic domain and a DEAD-box helicase has not been shown previously in any organism. It could be imagined that as structured RNA substrates are unwound by RhlB they could be passed directly to the active site of RNase E. In *E. coli*, RhlB is not directly bound to the catalytic domain of RNase E, although it clearly cooperates with the ribonuclease components of the degradosome in RNA turnover through its interactions with the RNase E CTD (30). The differences and similarities between the *C. crescentus* and *E. coli* degradosomes highlight the force of convergent evolution in the recruitment of RNA helicase activity to the machinery of RNA metabolism.

From the crystal structure of *Cc*NTD_1–274_ presented here it appears that the partially helical S1 insert occupies the area where RNA is bound in the *E. coli* RNase E structure (Figure 5). Negatively charged side-chains (specifically Asp 129, Glu 131 and Glu 132) could potentially mimic the negatively charged RNA substrate. There are corresponding insertions of various lengths in the S1 domain of RNase E from other gram-negative proteobacteria and plant homologues (Supplementary Figure S1) (31). The presence and positon of the S1 insert in *Cc*NTD suggests that the enzyme will interact with its RNA substrates differently to the *E. coli* enzyme. For instance, the S1 insert might undergo a pronounced conformational change to allow access to the active site. The functional importance of the S1 insert is highlighted by the finding that deletion of this region in the *Arabidopsis* RNase E inhibits enzymatic activity and impedes plant growth (6).

**Figure 6.**
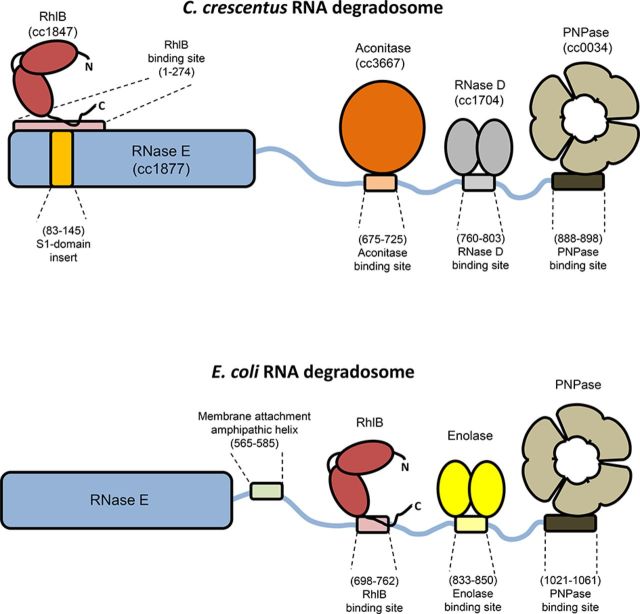
A schematic representation of the *Caulobacter crescentus* RNA degradosome in comparison to the paradigm degradosome assembly from *Escherichia coli*. Top: *C. crescentus* degradosome. The N-terminal domain of RNase E is shown as a solid blue bar, with the binding site for RhlB, and the S1 insert indicated. The disordered C-terminal domain is shown as a thin wavy blue line, with interaction sites for aconitase, RNase D and PNPase indicated. RhlB, aconitase, RNase D and PNPase are depicted as red, orange, grey and brown filled blocks respectively. Bottom: *E. coli* degradosome. As above, but with membrane attachment motif and enolase shown as green and yellow blocks respectively.

Based on the results reported here and from earlier studies (7) our current model of the protein interaction partners and binding sites in the *C. crescentus* RNA degradosome is summarized schematically and compared to the *E. coli* assembly in Figure 6. This comparison highlights the divergence between the two machines, including recruitment of different enzyme partners, location of the RNA helicase and the presence or absence of a membrane attachment motif. As in *E. coli*, the formation of a degradosome assembly is not essential for survival of *C. crescentus*, although its maintenance throughout evolution argues that it does impact on long term fitness (27,32). Our findings expand the current understanding of the protein components and modular interactions utilized by this multi-enzyme machine in the course of evolution. The molecular evolution of these machines in divergent species highlights their likely roles as multifaceted hubs of RNA metabolism and riboregulation (1).

## SUPPLEMENTARY DATA

Supplementary Data are available at NAR Online.

SUPPLEMENTARY DATA

## ACCESSION NUMBERS

PDB IDs: 4OXP.
