# Supplementary material for: Molecular recognition of RhlB and RNase D in the Caulobacter crescentus RNA degradosome
Source: Nucleic Acids Res. 2014 Nov 11;42(21):13294–305. doi: 10.1093/nar/gku1134 (PMC4245959; doi:10.1093/nar/gku1134)
Supplement: SUPPLEMENTARY DATA [file supp_42_21_13294__index.html]

Molecular recognition of RhlB and RNase D in the Caulobacter crescentus RNA degradosome — Molecular recognition of RhlB and RNase D in the Caulobacter crescentus RNA degradosome — SUPPLEMENTARY DATA 

# Molecular recognition of RhlB and RNase D in the *Caulobacter crescentus* RNA degradosome

## SUPPLEMENTARY DATA

**Files in this Data Supplement:**

- SUPPLEMENTARY DATA
