# Supplementary material for: Molecular recognition of RhlB and RNase D in the Caulobacter crescentus RNA degradosome
Source: Nucleic Acids Res. 2014 Nov 11;42(21):13294–305. doi: 10.1093/nar/gku1134 (PMC4245959; doi:10.1093/nar/gku1134)
Supplement: SUPPLEMENTARY DATA [file supp_gku1134_nar-02500-r-2014-File008.pdf]

Figure S1. Conservation and secondary structure prediction of the S1 and 5'-sensor domains of RNase E

(A) Multiple sequence alignment of the S1 and 5'-sensor domains of RNase E from divergent organisms. Representative α-proteobacteria (*Caulobacter crescentus, Brucella melitensis, Rhodobacter sphaeroides*), γ-proteobacteria (*Escherichia coli, Shigella flexneri, Erwinia tasmaniensis*), β-proteobacteria (*Vibrio cholerae, Pseudomonas aeruginosa, Burkholderia cepacia*) and plant (*Arabidopsis thaliana, Ricinus communis, Oryza sativa subsp. japonica*) species are shown. The S1 domains, S1 inserts (where present) and 5' phosphate sensor domains are indicated above the sequences.

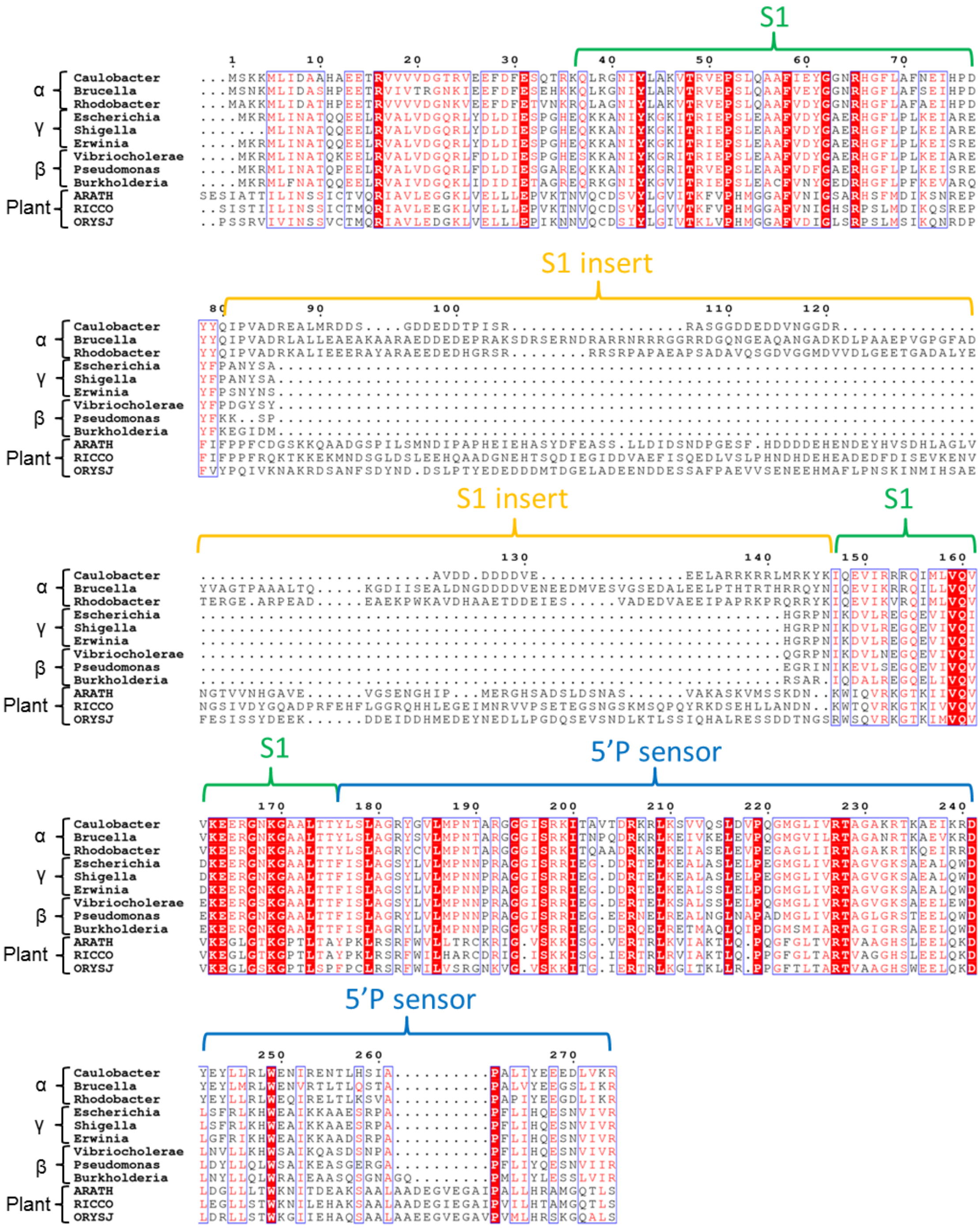

(B) PONDR prediction of structural disorder in the S1 and 5'-sensor domains. PONDR predicts intrinsically disordered regions of proteins based on amino acid sequence. Blue line indicates the PONDR prediction for the sequence of the S1 and 5'-sensor domains present in the *Cc*NTD$_{1\text{-}274}$ crystal structure.

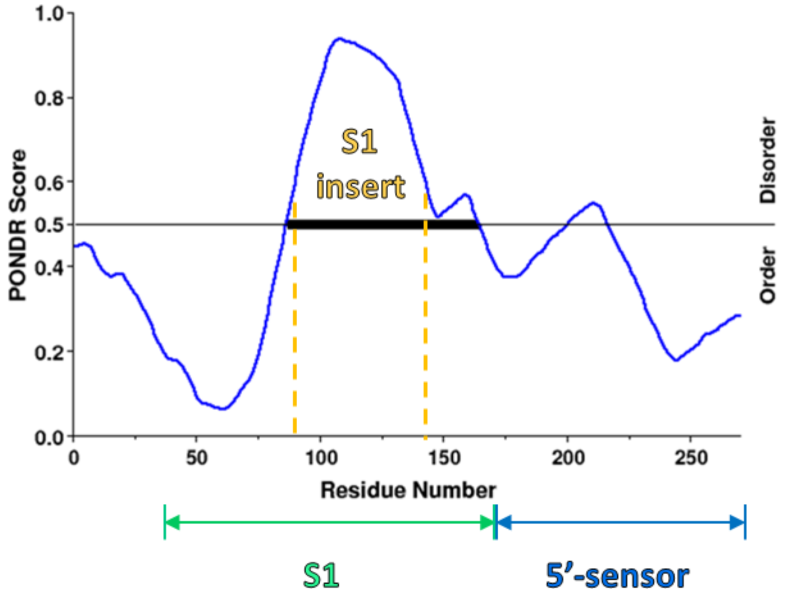

Figure S2. Co-immuno purification of *C. crescentus* RNase E and negative control. (Left lane) A representative pulldown of RNase E via an anti-RNase E antibody, showing the co-purification of the degradosome proteins Aconitase, PNPase, RhlB and RNase D. **(Right lane)** Negative control experiment, performed in the absence of anti-RNase E antibody. Significant non-specific enrichment of EftU is seen as a band running at an equivalent position to RNase D.

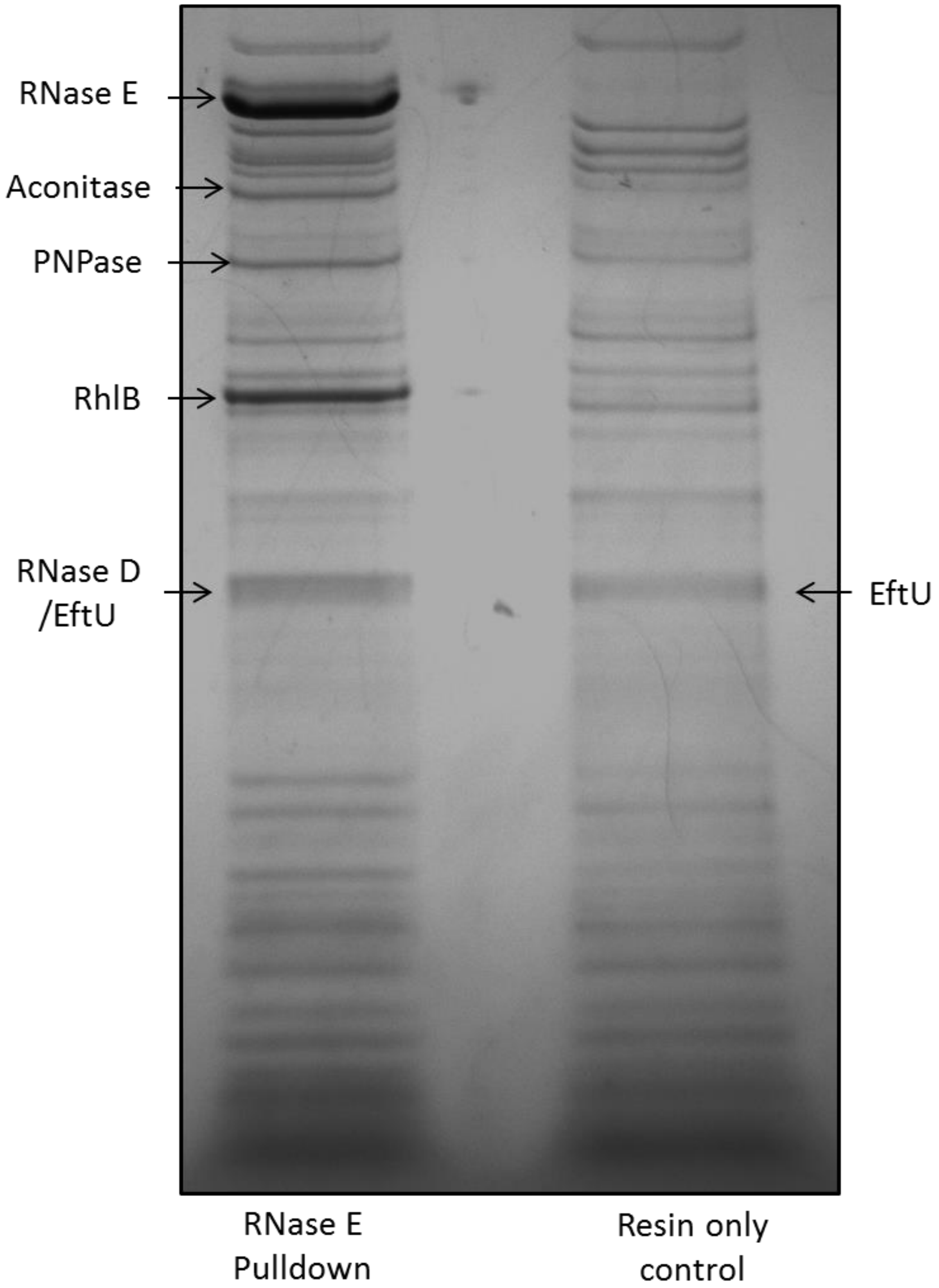

Figure S3. Pulldown of the *C. crescentus* RNA degradosome via RNase D (expanded version of Figure 1D). N-terminally His-tagged RNase D bait protein is shown as input lane. Known degradosome proteins (RNase E, Aconitase, PNPase and RhlB) are identified in the pulldown lane. A negative control is shown of the pulldown experiment in the absence of input RNase D, to highlight that multiple *C. crescentus* proteins bind non-specifically to the Nickel affinity resin at low levels. Particularly, a band running at the approximate position of RhlB in the negative control experiment was identified as the molecular chaperone GroEL by mass spectrometry, whereas the band at the equivalent position in the pulldown lane was identified as a mixture of RhlB and GroEL.

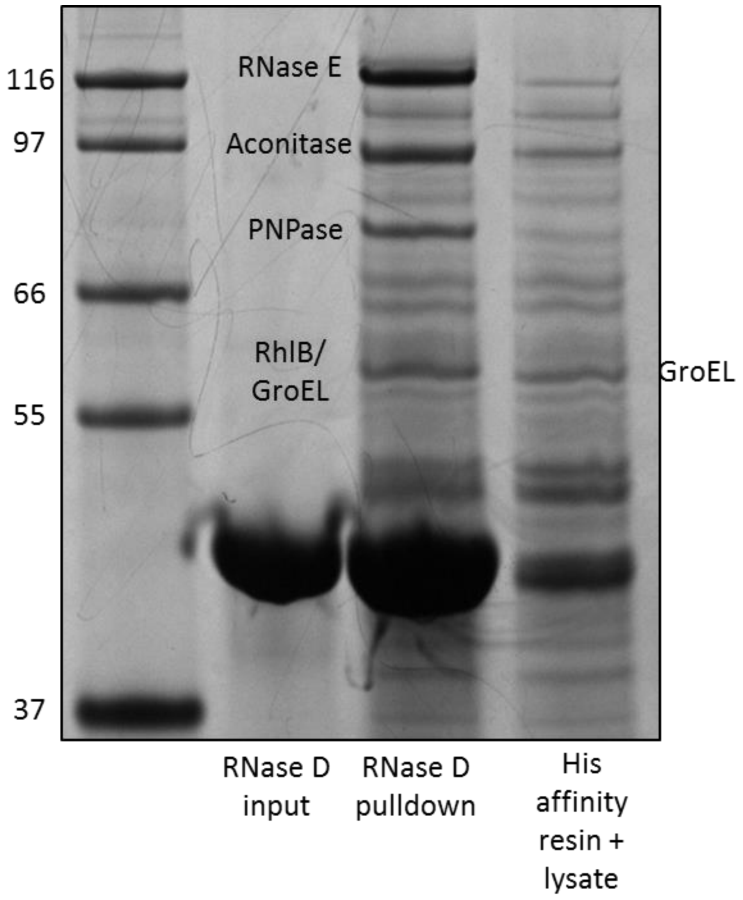

Figure S4. Sedimentation velocity analytical ultracentrifugation of *C. crescentus* RNase D. RNase D forms a dimer in solution. (A) UV280 Absorbance scans taken at 50,000 rpm. (B) Residuals from fitting the absorbance data to a continuous molecular mass distribution. (C) Continuous molecular mass distribution [c(M)] was fitted using SEDFIT. The peak value corresponds to an approximate mass of 88,900 Da (theoretical mass of RNase D dimer with N-terminal His-tag = 89,300 Da).

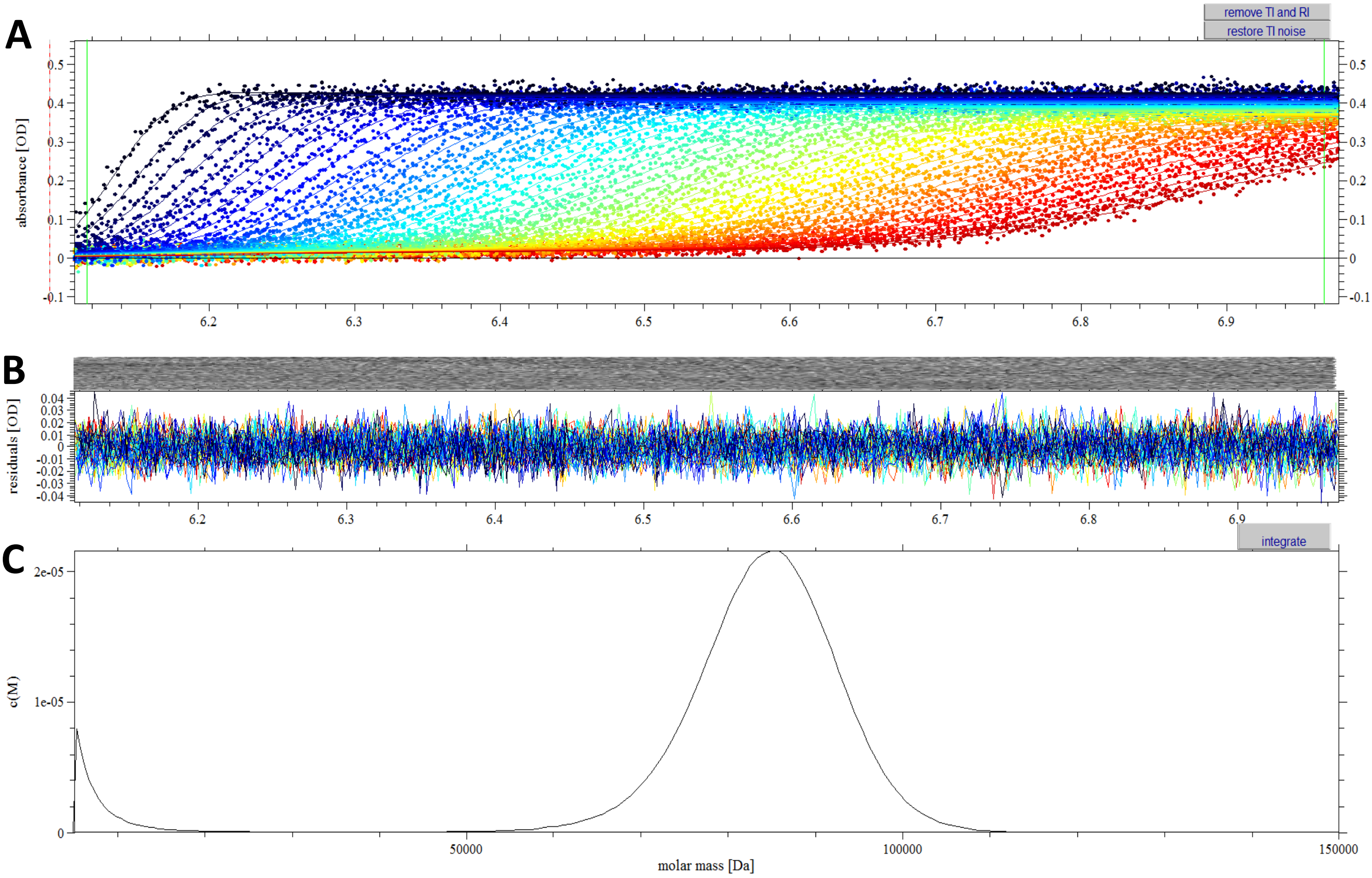

Figure S5. Sedimentation velocity analytical ultracentrifugation of the *Cc*NTD – RhlB complex . *Cc*NTD forms a 1:2 complex with RhlB (tetramer *Cc*NTD:monomer RhlB) in solution. (A) UV280 Absorbance scans taken at 40,000 rpm for the complex (2 mg/mL). (B) Residuals from fitting the absorbance data to a continuous molecular mass distribution. (C) Continuous molecular mass distribution [c(M)] was fitted using SEDFIT. The peak value corresponds to an approximate mass of 380,000 Da (theoretical mass of 1:2 complex = 370,000 Da).

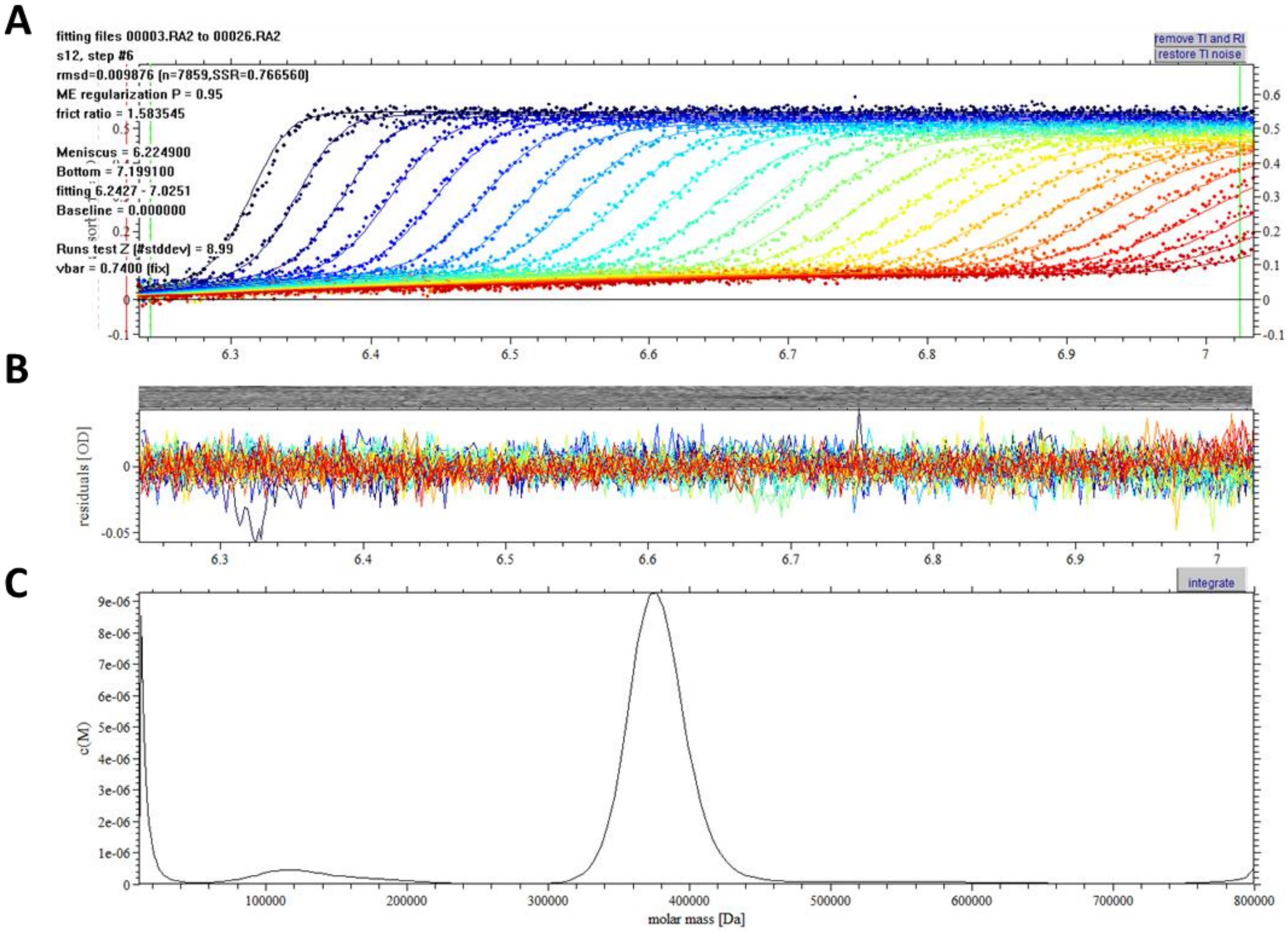

Figure S6. Sedimentation velocity analytical ultracentrifugation of CcNTD. CcNTD forms a tetramer in solution. (A) Interference scans taken at 40,000 rpm for CcNTD (2 mg/mL). (B) Residuals from fitting the interference data to a continuous molecular mass distribution. (C) Continuous molecular mass distribution [c(M)] was fitted using SEDFIT. The peak value corresponds to an approximate mass of 265,000 Da (theoretical mass of tetramer = 260,000 Da).

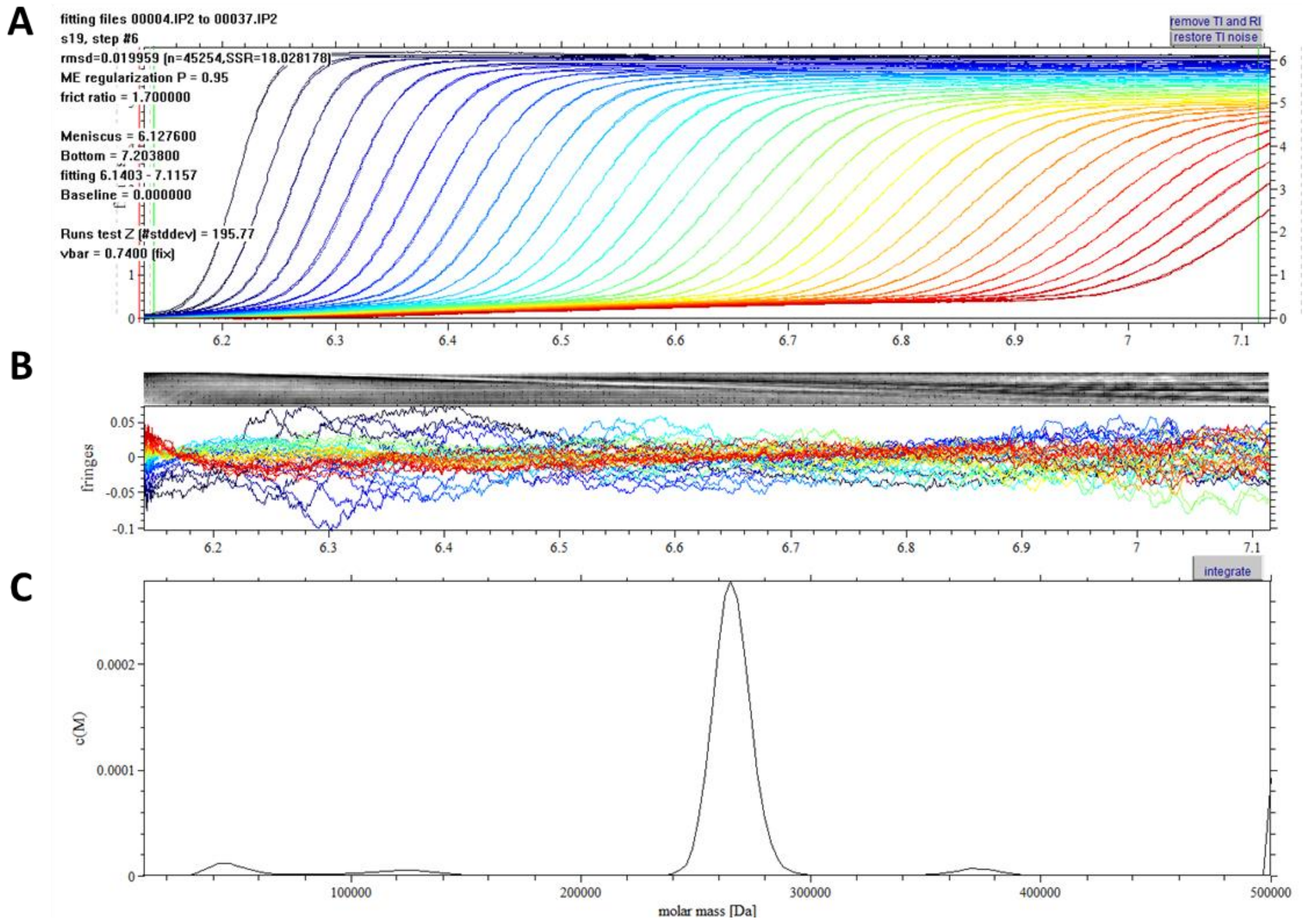

Figure S7. Sedimentation velocity analytical ultracentrifugation of CcRhlB. CcRhlB forms a monomer in solution. (A) Interference scans taken at 40,000 rpm for CcRhlB (2 mg/mL). (B) Residuals from fitting the interference data to a continuous molecular mass distribution. (C) Continuous molecular mass distribution [c(M)] was fitted using SEDFIT. The peak value corresponds to an approximate mass of 56,900 Da (theoretical mass of monomer = 56,700 Da).

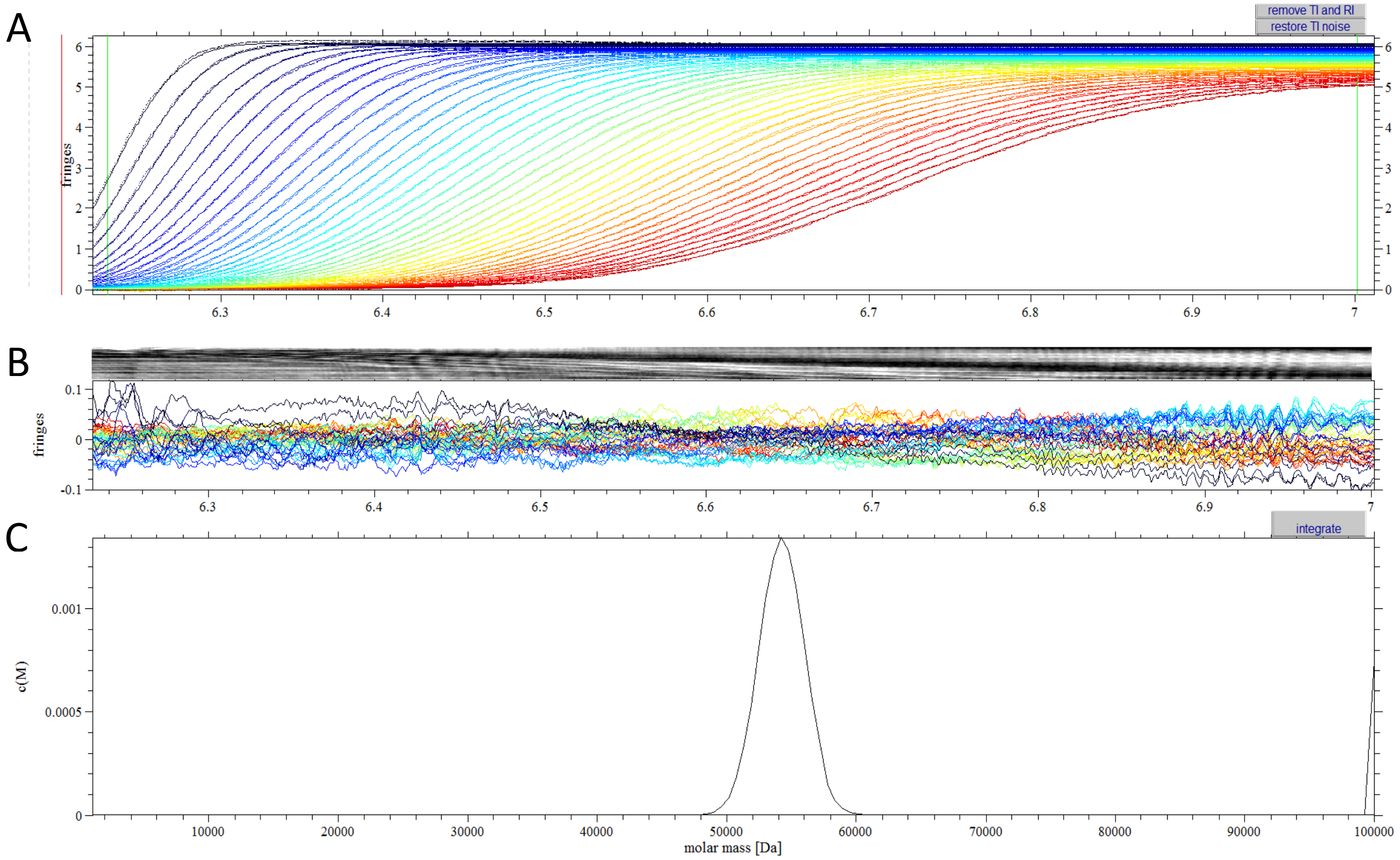

Figure S8 Sedimentation velocity analytical ultracentrifugation of CcNTD1-274 – RhlB complex
CcNTD1-274 forms a 1:1 complex with RhlB in solution. (A) Interference scans taken at 50,000 rpm for CcNTD1-274 – RhlB (2 mg/mL). (B) Residuals from fitting the interference data to a continuous molecular mass distribution. (C) Continuous molecular mass distribution [c(M)], which was fitted using SEDFIT. The peak value corresponds to an approximate mass of 93,000 Da (theoretical mass of a 1:1 complex = 88,000 Da).

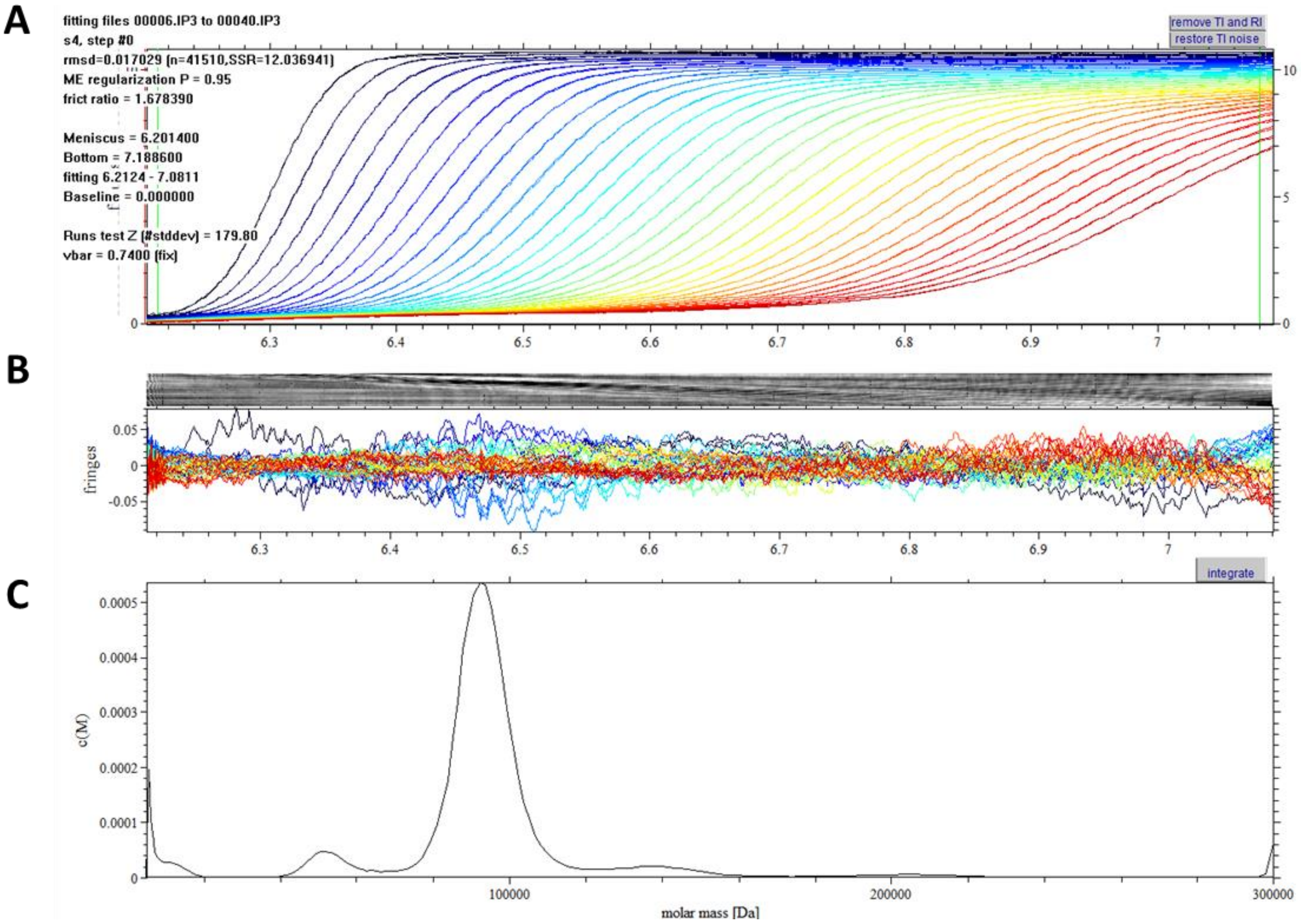

Figure S9. ANCHOR prediction of RhlB. Red line indicates probability of structural disorder according to the IUPred algorithm. Blue line is the ANCHOR score for predicted protein – protein interaction sites.

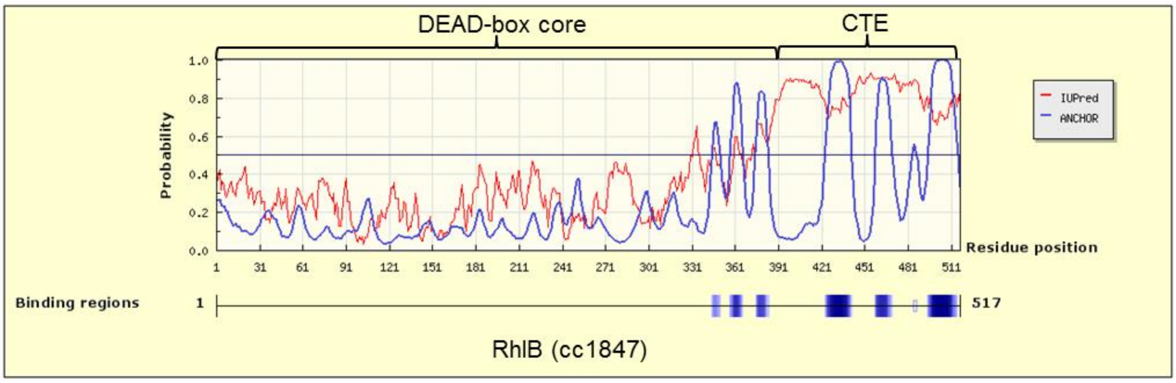

Figure S10. Sequence alignment of RhlB. Sequences from *E. coli* and *C. crescentus* are aligned with two homologs, Vasa from *Drosophila* and Mss116p from yeast, whose crystal structures are known. The helicase core domain and C-terminal extension are indicated.

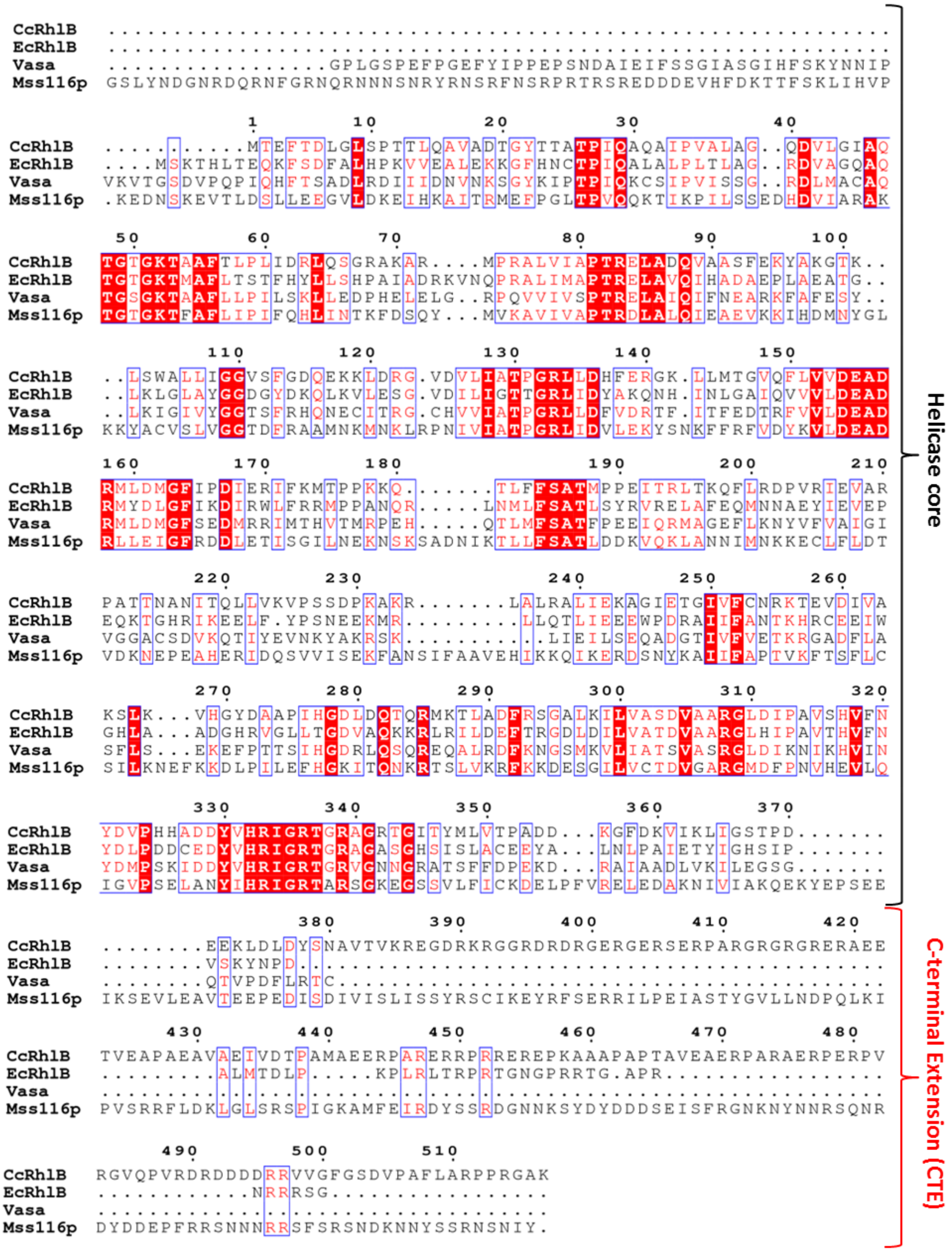

Figure S11. Sequence alignment of CcNTD and EcNTD. The entire RNase E catalytic domain sequence is shown. (CcNTD = 1-575, EcNTD = 1-527). The region involved in the interaction with RhlB ($CcNTD_{1-274}$) is shown in the green boxed area.

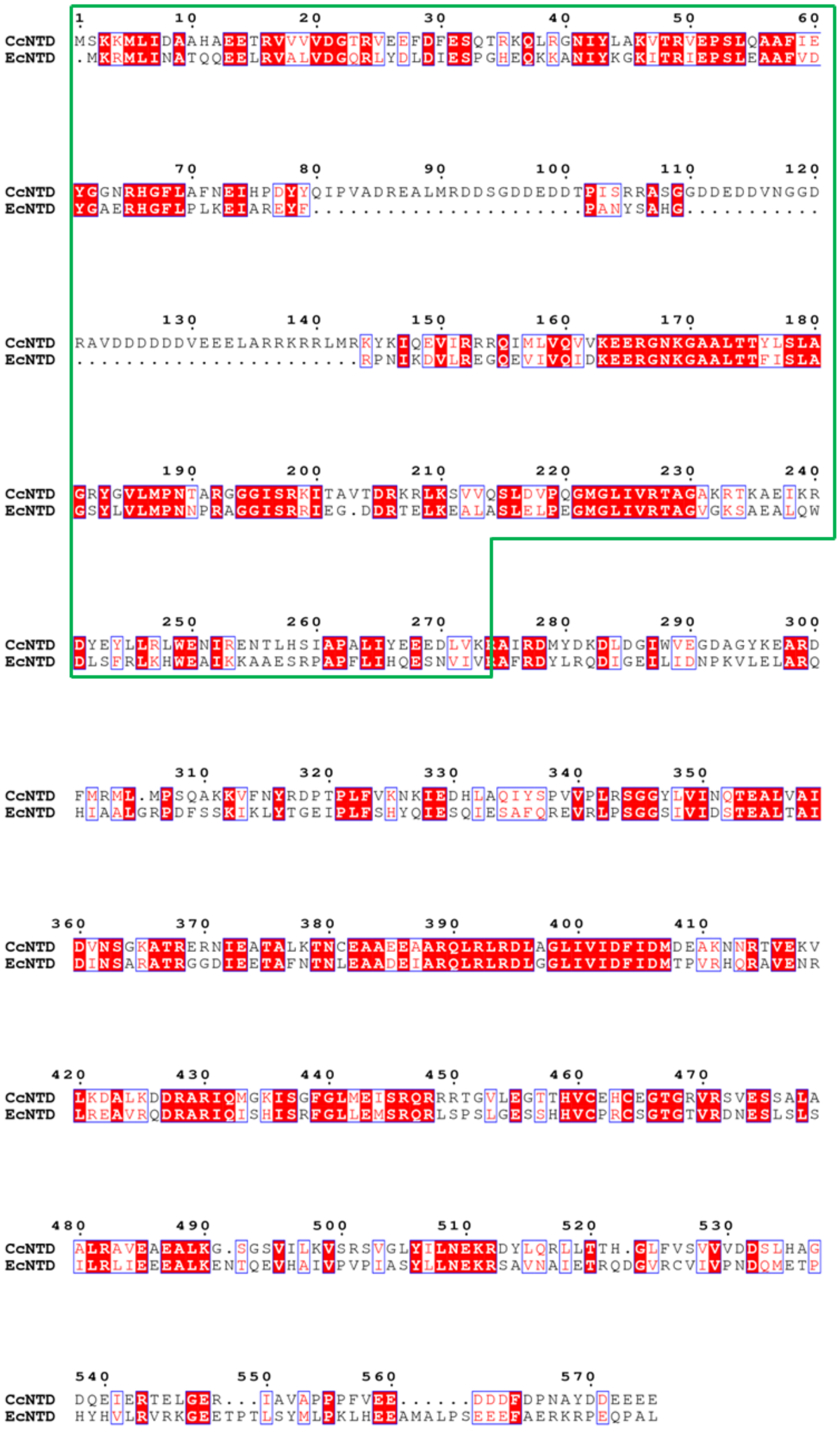

Figure S12. Sequence alignment of *E. coli* and *C. crescentus* RNase D proteins. Coli = *E. coli* RNase D, CCrnd1 = *C. crescentus* protein cc1704 (RNase D present in the RNA degradosome), CCrnd2 = *C. crescentus* protein cc3603 (truncated RNase D, not present in the RNA degradosome).

```
       1        10        20        30        40        50
Coli   MNYQMITTDDALASLCEAVRAFPAIALDTEFVRTRTYYPQLGLIQLFDGEHLALIDPLG.
CCrnd1 ..MKLITTTAELAAFCNGLAGEPFVAVDTEFMRETTYWPKLCLIQVASPTQEAVIDPLAD
CCrnd2 ..MANFVHEGDLP..DGVFADAEIIAIDSETMGLRLRRDPLCVVQLSAGDGDAHVVRLSR

       60        70        80        90        100       110
Coli   .ITDWSPLKAILRDPSITKFLHAGSEDLEVFLNVFGELPQPLIDTQILAAFCG.RPMSWG
CCrnd1 .NIDLEPLLVVMRDERILKVFHAARQDVEIFNNLK.AMPKPLFDTQVAGMAAG.FGEQIA
CCrnd2 PDYDCPNLKALLTNPKVLKLFHFGRFDIAMFELHLGVVTAPVYCTKIASKLARTYTDRHG

       120       130       140       150       160       170
Coli   FASMVEEYSGVTLDKSESRTDWLARPLTERQCEYAAADVWYLLPITAKLMVETEASGWLP
CCrnd1 YDALVRQMLRIEIDKSSRFTDWARRPLTDAQLSYALADVTHLAKLFPILRERLETSGRLA
CCrnd2 LKDVTRELLGVELSKVQQSSDWGAPSLSVDQVAYAASDVLHLHGLRLKLNAMLEREGRMA

       180       190       200       210       220       230
Coli   AALDECRLMQMRRQEVVAPEDAWRDITNAWQLRTRQLACLQLLADWRLRKARERDLAVNF
CCrnd1 WVEEEMTAISDPAAYDVDPEKAWRRLR.PRKTAPKYLAVFKAVAAWRERTAQNRDQPRGR
CCrnd2 LAQHAFDYLPHRASLDLAG...WEEMDIFAHS............................

       240       250       260        270       280       290
Coli   VVREEHLWSVARYMPGSLGELDSLG..LSGSEIRFHGKTLLALVEKAQTLPEDALPQPML
CCrnd1 ILKDEAIDELATQTPGNLEALNNLRGVPKGFGGSKFGPDLLAAIKDALADPEGYAPVVDK
CCrnd2 ............................................................

          300        310       320       330                 340
Coli   NLMDMPGYR.KAFKAIKSLITDVSETHKISAELLASRRQINQLLN.........WHWKLK
CCrnd1 PGPPPPQNAGAVVELLKVLLKARAEEAGVASKLIATVSDLEKIAADDNATTPALTGWRRE
CCrnd2 ............................................................

          350       360       370
Coli   PQNNLPELISGWRGELMAEALHNLLQEYPQ.....
CCrnd1 AFGSDALKIKRGELALVLDGARVRVVEVRRAPKND
CCrnd2 ...................................
```
